# Community utilisation and satisfaction with the community-based health planning and services initiative in Ghana: a comparative study in two system learning districts of the CHPS+ project

**DOI:** 10.1186/s12913-020-05678-5

**Published:** 2020-09-09

**Authors:** Margaret Kweku, Hubert Amu, Martin Adjuik, Fortress Yayra Aku, Emmanuel Manu, Elvis Enowbeyang Tarkang, Joyce Komesuor, Geoffrey Adebayor Asalu, Norbert Ndaah Amuna, Laud Ampomah Boateng, Justine Sefakor Alornyo, Roland Glover, Ayaga A. Bawah, Timothy Letsa, John Koku Awoonor-Williams, James F. Phillips, John Owusu Gyapong

**Affiliations:** 1 School of Public Health, University of Health and Allied Sciences, Hohoe, Ghana; 2 Volta Regional Health Directorate, Ghana Health Service, Ho, Ghana; 3 Regional Institute of Population Studies, University of Ghana, Legon, Ghana; 4 Policy Planning Monitoring and Evaluation Division, Ghana Health Service, Accra, Ghana; 5 Mailman School of Public Health, Columbia University, New York, USA; 6 Office of the Vice-Chancellor, University of Health and Allied Sciences, Ho, Ghana

**Keywords:** CHPS services, Utilisation, Satisfaction, Community members, System Learning District, Nkwanta South Municipality, Central Tongu District, Volta Region, Ghana

## Abstract

**Background:**

To strengthen the implementation of the Community-based Health Planning and Services (CHPS) programme which is Ghana’s key primary health care delivery strategy, the CHPS+ Project was initiated in 2017. We examined community utilisation and satisfaction with CHPS services in two System Learning Districts (SLDs) of the project.

**Methods:**

This community-based descriptive study was conducted in the Nkwanta South Municipality and Central Tongu District of Ghana. Data were collected from 1008 adults and analysed using frequency, percentage, chi-square, and logistic regression models.

**Results:**

While the level of utilisation of CHPS services was 65.2%, satisfaction was 46.1%. Utilisation was 76.7% in Nkwanta South and 53.8% in Central Tongu. Satisfaction was also 55.2% in Nkwanta South and 37.1% in Central Tongu. Community members in Nkwanta South were more likely to utilise (AOR = 3.17, 95%CI = 3.98–9.76) and be satisfied (AOR = 2.77, 95%CI = 1.56–4.90) with CHPS services than those in Central Tongu. Females were more likely to utilise (AOR = 1.75, 95%CI = 1.27–2.39) but less likely to be satisfied [AOR = 0.47, 95%CI = 0.25–0.90] with CHPS services than males. Even though subscription to the National Health Insurance Scheme (NHIS) was just 46.3%, NHIS subscribers were more likely to utilise (AOR = 1.51, 95%CI = 1.22–2.03) and be satisfied (AOR = 1.45, 95%CI = 0.53–1.68) with CHPS services than non-subscribers.

**Conclusion:**

Ghana may not be able to achieve the goal of universal health coverage (UHC) by the year 2030 if current levels of utilisation and satisfaction with CHPS services persist. To accelerate progress towards the achievement of UHC with CHPS as the vehicle through which primary health care is delivered, there should be increased public education by the Ghana Health Service (GHS) on the CHPS concept to increase utilisation. Service quality should also be improved by the GHS and other stakeholders in Ghana’s health industry to increase satisfaction with CHPS services. The GHS and the National Health Insurance Authority (NHIA) should also institute innovative strategies to increase subscription to the NHIS since it has implications for CHPS service utilisation and satisfaction.

## Background

Globally, critical public health concerns like morbidity and health inequity result from disparities in the availability, access, and utilisation of health services especially in low- and middle-income countries (LMICs) [1]. In response, the 1978 international conference on Primary Health Care (PHC) was organised at Almaty (formerly Alma-Ata) [2]. At the conference, PHC was identified as the key to attaining Universal Health Coverage (UHC) which was defined as essential health care made universally accessible to individuals and acceptable to them, through their full participation and at a cost that the community could afford [2]. This implies that the proximity, acceptance, satisfaction and perceived relevance of facilities within the catchment of communities, especially rural ones, is important in achieving UHC [3, 4]. The Sustainable Development Goal (SDG) 3 which seeks to ensure healthy lives and promote wellbeing for all at all ages by the year 2030 [5, 6] is one of the strategies geared towards achieving UHC in LMICS.

Pursuant to the Almaty declaration, Ghana implemented various interventions towards achieving UHC. One of such interventions is the Community-based Health Planning and Services (CHPS) programme which was initiated in 1999 [7, 8]. The CHPS concept is a national health system initiative aimed at providing accessible primary health care through the reduction of physical and geographical limitations to health care accessibility in deprived communities in Ghana [9]. The role of CHPS in PHC implementation is thus to bring health services to local communities, develop sustainable volunteerism and community health action, empower vulnerable populations such as women, and enhance healthcare provider-community interaction [7]. Though not to desirable levels, the implementation of CHPS has led to reduced water-borne diseases, reduced infant and child mortalities through immunization, increased household involvement in the treatment of diarrhoea, and breastfeeding [9–11].

Even though it is recognised that community utilisation and satisfaction with health services are important indicators of health systems performance and are useful tools in predicting future service utilisation [12, 13], the utilisation and satisfaction with CHPS services have seen decreasing trends over past two decades [14–16] because CHPS services have been focused on the provision of clinical services and the construction of infrastructure to the detriment of community-based approaches which ensure that CHPS services are sent to the doorsteps of communities [17]. There was, therefore, the need to introduce interventions to improve its implementation and increase community utilisation and satisfaction with CHPS services. The result was the introduction of a National Program for Strengthening the Implementation of CHPS Initiative in Ghana (CHPS+) from which the current study was conducted. The purpose of the study was, therefore, to understand the level of community utilisation and satisfaction with CHPS services as part of the CHPS+ Project. Findings from the research will inform policymakers on measures needed to increase community ownership of CHPS as the key PHC intervention and its overall performance in improving health outcomes in Ghana.

### The CHPS+ project

CHPS+ is a scale-up of the Ghana Essential Health Interventions Programme (GEHIP) [8]. It is a collaboration project by the GHS, the Mailman School of Public Health of the Columbia University, USA, the University of Ghana’s Regional Institute of Population Studies, Ghana, the University of Health and Allied Sciences’ (UHAS) School of Public Health (SPH), and the University for Development Studies (UDS) with funding from the Doris Duke Charitable Foundation, USA. As part of the project implementation, UHAS and UDS are to develop the capacity of frontline health workers through the development of short- and long-term training programmes in two administrative regions; Volta and Northern Regions respectively.

In the Volta Region, the project primarily targets two administrative districts (Nkwanta South Municipality and Central Tongu District) selected by the regional health directorate of the GHS and labelled ‘System Learning Districts’ (SLDs). This, therefore, influenced the choice of the two districts for our study. The Volta Region was selected for the project as a result of having one of the highest under-five mortalities (61/1000) and fertility (4.8) rates in Ghana [4]. UHAS, the institution which led the current research is the project partner mandated to develop the capacity of frontline health workers in the Volta Region. The choice of UHAS for the project was due to its focus on problem-based learning approach (christened ‘vocational training programme’) adopted in training students upon its establishment in 2011 by Act 828 of the Parliament of Ghana (became operational in 2012) [18].

The CHPS+ project has three implementation phases required for UHAS. Phase I comprises engaging in consultative meetings with the SLDs while Phase II entails planning and conducting stakeholder (frontline health workers and community members) engagements in the SLDs to identify frontline health workers’ (CHOs, the staff at the sub-district level, and those at the district level) capacity development needs. The goal of phase II after identifying the training needs is to develop short and long-term capacity development curricular to help strengthen the implementation of CHPS by training the frontline health workers on the developed curricular.

To achieve the phase II expectations of the project after successfully executing the phase I, the university conducted a stakeholder engagement using a participatory approach to assess the capacity development needs of frontline health workers in the two SLDs. The result of that engagement was the development of a training curriculum which comprised 16 courses further divided into five modules. In August 2018, the university conducted training for the frontline workers on the Module One (UHAS+ 101: Community Entry and Organisation; UHAS+ 102: Cultural Competency in Health Care Delivery; and UHAS+ 103: Communicative Skills [Professional Communication]) of the curriculum in the two SLDs. At the end of the training which took place from 6th – 11th and 20th – 25th August respectively in Nkwanta South and Central Tongu, some common action points emanated which the trained frontline health workers and their respective district/municipal health directorates were expected to implement as the project is based on the implementation research approach. The action points included the need to increase community utilisation and satisfaction with CHPS services. With these action points in mind, 3 months after the training, the UHAS team conducted monitoring in selected communities in the two SLDs. The current research is the result of that monitoring which was conducted from 9th – 13th December 2018 in Nkwanta South and from 16th–20th December 2018 in Central Tongu. This study focuses on the part of the monitoring concerning community utilisation and satisfaction with CHPS services.

## Methods

### Study setting

This study was conducted in Central Tongu and Nkwanta South SLDs of the CHPS+ Project. Nkwanta South and Central Tongu are two of the 25 administrative districts in the Volta Region [19]. While Central Tongu is located in the southern coastal part of the region, Nkwanta South is situated at the Northern savannah belt [19]. The Volta Region is also one of the ten administrative regions in Ghana [19]. The region’s total land area is 20,570 km^2^, representing 8.7% of the total land area of Ghana. The region has a total population of 2,549,256 with a growth rate of 2.5% as projected from the 2010 national population census. Out of this, males constituted 1,252,936 [19, 20].

**Fig. 1:**
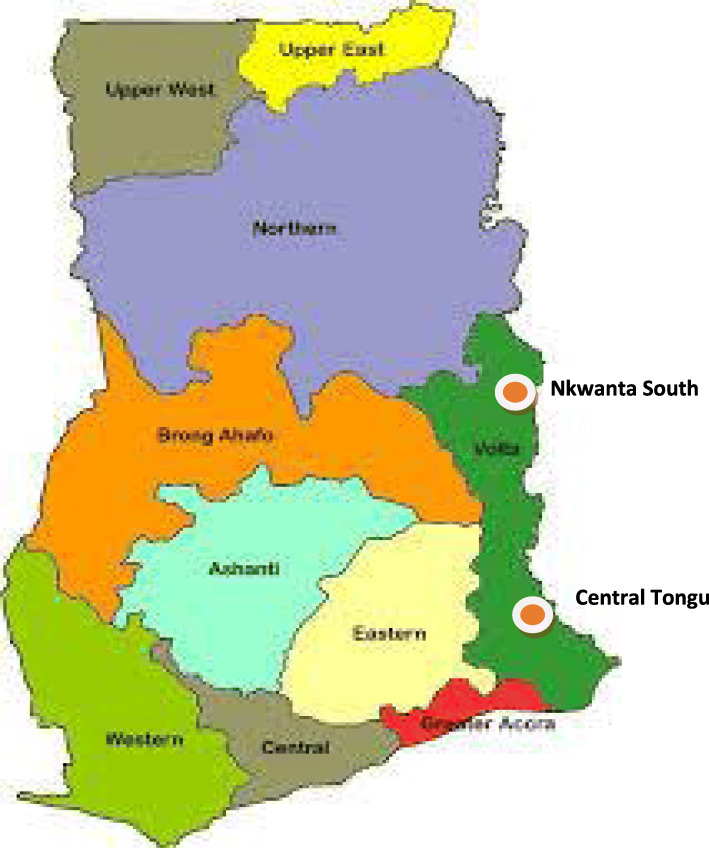
Map of Study setting. Source: Created by the Authors

The Central Tongu District has a total population of 71,302 a growth rate of 2.4% and the population covered by CHPS is 38,874 (54.5%). The district has 500 communities with Adidome being the district capital. There is one district hospital, 4 health centres, and 1 private maternity home. The district has 18 demarcated CHPS zones out of which 15 zones are functional. The Nkwanta South Municipality has a population of 142,602 with a growth rate of 3%. There are 2 hospitals, 2 health centres and 1 maternity home. There are also 25 demarcated CHPS zones with 21 of them being functional CHPS zones [19]. A functional CHPS zone is one which has a CHPS compound which operates to support or facilitate emergency referrals besides the daily home visits and outreach activities [4]. Figure 1 presents a map of the study setting.

### Population and design

A community-based descriptive cross-sectional study was carried out among adults aged 18 years and above in the two SLDs in December 2018. Community members were assessed using a pretested interviewer-administered semi-structured questionnaire. The assessment included utilisation and satisfaction with CHPS. Residents who had lived in the community for at least 6 months belonged to a household and consented were recruited to participate in the study.

### Sampling

The sample size for the study was calculated using the Cochran [21] formula for sample size determination. The formula is given as n = $\frac{{\left(\mathrm{Z}\upalpha /2\right)}^2\left(1-p\right)p}{d^2}$, The sample size for the study is determined below.

$$ \mathrm{n}=\frac{{\left(\mathrm{Z}\upalpha /2\right)}^2\left(1-p\right)p}{d^2}\kern0.5em =\frac{1.962\ x\ 0.50\ x\ \left(1-0.50\right)}{0.052}=384.16=385 $$

Where: n = Required sample size, Z = Confidence level at 95% (standard value of 1.96), P = Prevalence in the population (50% default value for this study), d = Level of Precision/margin of error of 0.05. Assuming a non-response rate of 10% of the sample size gives 38.5 = 39. This gives a total of 424. Thus, 405 for each district making a total sample of 848. However, a total of 1008 participants were studied, 504 from each study district.

A two-stage cluster sampling technique was used in selecting respondents for the study. The first stage involved the selection of communities. With this, six communities each were randomly selected out of the functional CHPS zones of the two clusters (the SLDs) using lottery method. At this stage, all the names of the communities in the functional zones of each SLD were written on pieces of paper. The papers were wrapped into small balls, placed in a container, and the container was thoroughly shaken. The first community was then selected. This process was repeated until the last community was selected in each district.

The WHO cluster random sampling was used at the community level to select participants. In the selection of households, the centre of the community was located, and a random household was chosen by spinning a bottle. The first household in the direction where the bottle pointed then became starting household surveyed. Every other 5th household in that direction was then included in the data collection. The number 5 was used based on the sampling fraction derived. A household is defined as a group of people who live under the same roof and eat together. In each household, a male, female, or both (18+ years of age) were interviewed once present.

### Data collection

We developed a structured questionnaire and used it as the main instrument for data collection (See Additional File 1). The questionnaire was divided into three sections; 100, 200, and 300. The first section focused on the socio-demographic characteristics of the respondents. They were age, sex, religion, marital status, ethnicity, educational level, occupation, average monthly income, and National Health Insurance Subscription status. The second section focused on CHPS services utilisation among community members and was made up of seven variables; home visit, family planning, antenatal care, skilled delivery, post-natal (PNC) services, child welfare clinic and immunisation as well as treatment of minor ailments. The respondents were asked to select the various CHPS services they utilised in the 6 months preceding the survey. The third section also focused on community members’ satisfaction with the CHPS services received in the second section. In this section, respondents were required to indicate their level of satisfaction with the services on a Likert scale of very satisfied (coded as 4), satisfied (coded as 3), not satisfied (coded as 2) and not at all satisfied (coded as 1). An overall dichotomous satisfaction index variable of satisfied (1) and not satisfied (0) was computed using the composite scores of the various variables. To achieve the index variable, a mean scored of 11.08 was generated as the cut-off point. Those who scored above the mean were considered ‘satisfied’. This variable was then used in running the logistic regression analyses. Reliability analysis was done to determine the appropriateness of the items of the instrument in measuring the expected outcomes, using a Cronbach’s alpha analysis. A Cronbach’s alpha coefficient of 0.90 derived indicated that the instrument was reliable based on Taber’s interpretation [22].

Eighteen research assistants from the School of Public Health (SPH) of UHAS were trained as field assistants for 2 days on the data collection tools. Four lecturers from SPH-UHAS also served as the supervisors of these field assistants. During the training, the field assistants were informed about the purpose of the study and taken through all the data collection tools. The training was done to ensure that all data collectors understood how to ask questions to enable the respondents to provide appropriate answers. On the second day of the training, pre-testing of the instruments was done in one community in the Hohoe Municipality which is also one of the 25 administrative districts of the Volta Region. This was done to enable the research team to know how the respondents understood and responded to the questions. No major changes were, however, done on the tools after the pre-testing.

Permission for the data collection was sought from the health directorates of the two SLDs through letters. Meetings were also held with opinion leaders of the sampled communities to seek their consent for the study. Informed consent was sought from each participant before questionnaires were administered. Names of participants and their communities were not collected in the questionnaires instead, codes were used as unique identifiers.

### Data analysis

Filled questionnaires were checked for completeness and corrected on the field. The data were then entered into Epi-data version 3.1 and exported into Stata/SE version 14.0 for analysis. Continuous variables were reported as mean ± standard deviation. Categorical variables were reported as proportions. Chi-square test was used to determine the association between variables of interest. Statistical significance was considered at *p* < 0.05. Logistic regression models were used to determine the strength of associations among variables of interest. Two regression models (I [bivariable] and II [multivariable]) were done.

## Results

### Socio-demographic characteristics of respondents

Table 1 presents the socio-demographic characteristics of respondents. A total of 1008 respondents from Central Tongu and Nkwanta South SLDs were involved in the study. The mean age was 39.2 ± 14.65. The majority (56.3%) of respondents were females. Most (81.8%) were Christians and married/Co-habiting (81.0%). Ewes were the most (64.4%) represented ethnic group in the two SLDs. Respondents constituting 34.4% attained Junior High School (JHS) level of education, while 28.4% had no formal education. Majority of the respondents were farmers (62.1%). The average monthly income of 60.3% of our respondents ranged from GH¢100–499. GH¢ (The Ghana Cedi) is the official currency of Ghana. Respondents who were subscribers to the NHIS also constituted 46.3%.

**Table 1:** Background Characteristic of respondents

Variable	District		Total
	Central Tongu [*N* = 504] n (%)	Nkwanta South [*N* = 504] n (%)	[*N* = 1008] N (%)
**Sex**			
Male	231(45.8)	210(41.7)	441(43.7)
Female	273(54.2)	294(58.3)	567(56.3)
**Mean age (in years)** 39.2 ± 14.65			
**Age group (in years)**			
< 30	156(31.0)	140(27.8)	296(29.4)
30–39	135(26.8)	135(26.8)	270(26.8)
40–49	95(18.8)	107(21.2)	202(20.0)
50+	118(23.4)	122(24.2)	240(23.8)
**Religion**			
Christianity	451(89.5)	373(74.0)	824(81.7)
Islam	19(3.8)	26(5.2)	45(4.5)
African Traditional	34(6.7)	105(20.8)	139(13.8)
**Marital Status**			
Married/Co-habiting	404(80.1)	413(81.9)	817(81.0)
Divorced/Separated	17(3.4)	21(4.2)	38(3.8)
Widowed	16(3.2)	27(5.4)	43(4.3)
Never married	67(13.3)	43(8.5)	110(10.9)
**Ethnicity**			
Ewe	496(98.4)	154(30.5)	650(64.5)
Akan	4(0.8)	31(6.2)	35(3.5)
Guan	1(0.2)	258(51.2)	259(25.7)
Others	3(0.6)	61(12.1)	64(6.3)
**Educational level**			
None	109(21.6)	177(35.1)	286(28.4)
Primary	107(21.2)	90(17.8)	197(19.5)
JHS	191(37.9)	156(31.0)	347(34.4)
SHS	70(13.9)	62(12.3)	132(13.1)
Tertiary	27(5.4)	19(3.8)	46(4.6)
**Occupation**			
Unemployed	40(7.9)	35(6.9)	75 (7.4)
Trader	65(12.9)	74(14.7)	139 (13.7)
Farmer	319(63.3)	307 (60.9)	626 (62.1)
Tradesman	27(5.4)	39 (7.7)	66 (6.6)
Seamstress/hair dresser	24(4.8)	27 (5.4)	51(5.1)
Civil servant	29(5.7)	22(4.4)	51(5.1)
**Income (in GHC)**			
Very low (< 100)	109(21.6)	129(25.6)	238(23.7)
Low (100–499)	297(59.2)	310(61.5)	607(60.3)
High (500–999)	60(12.0)	46(9.1)	106(10.5)
Very high (≥1000)	36(7.2)	19(3.8)	55(5.5)
**NHIS Status**			
Subscriber	224(44.4)	243(48.2)	467(46.3)
Non-subscriber	280(55.6)	261(51.8)	541(53.7)

### Utilisation of CHPS services

**Fig. 2:**
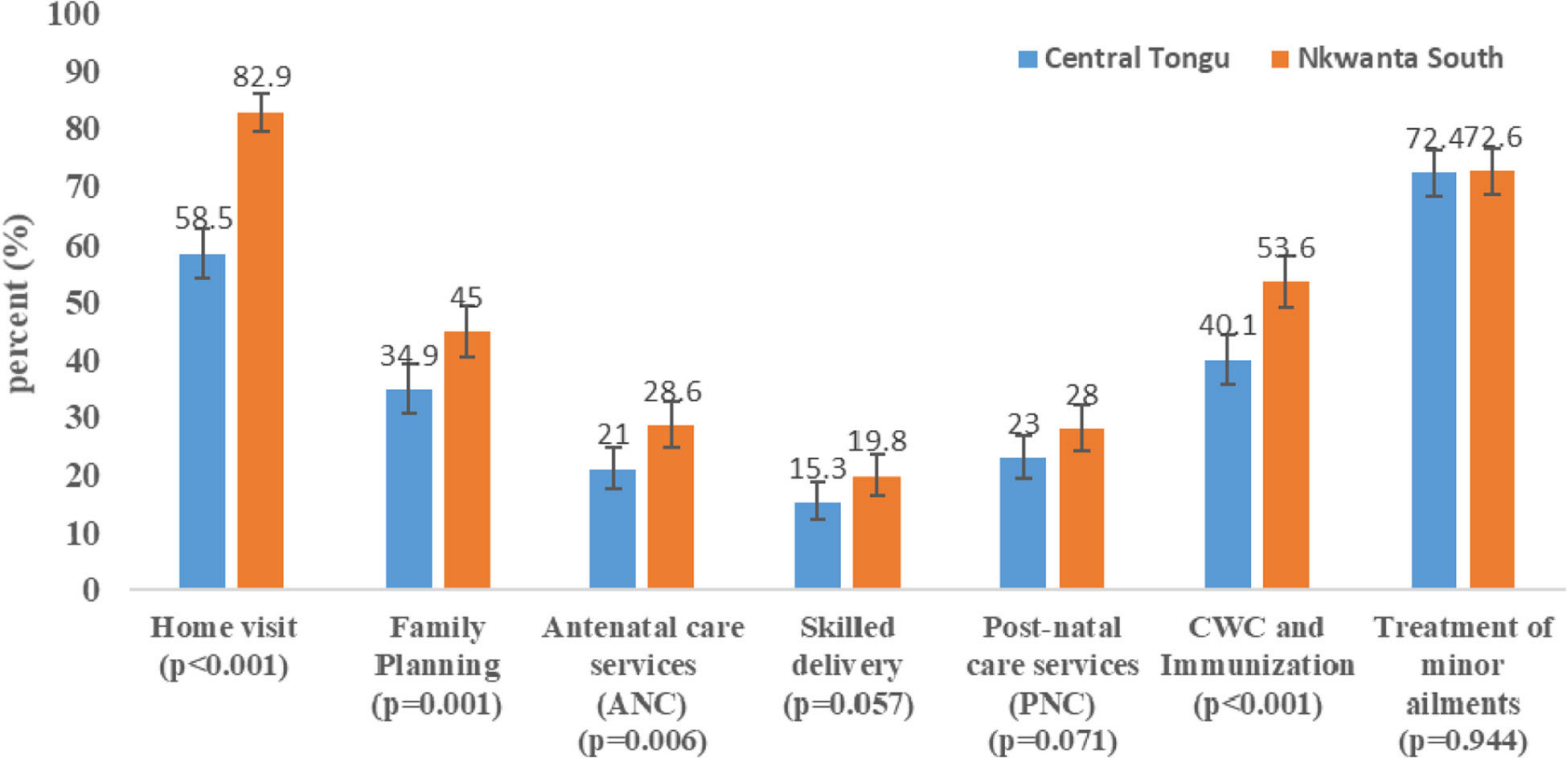
Level of Utilisation of various CHPS services by district

Figure 2 presents the CHPS services utilisation by respondents in the two SLDs. The utilisation of home visits, Family Planning (FP), Antenatal Care (ANC), as well as Child Welfare Clinic (CWC) and immunisation services were significantly higher in Nkwanta South than Central Tongu (*p* < 0.001, *p* < 0.01, *p* < 0.01, and *p* < 0.001) respectively.

**Fig. 3:**
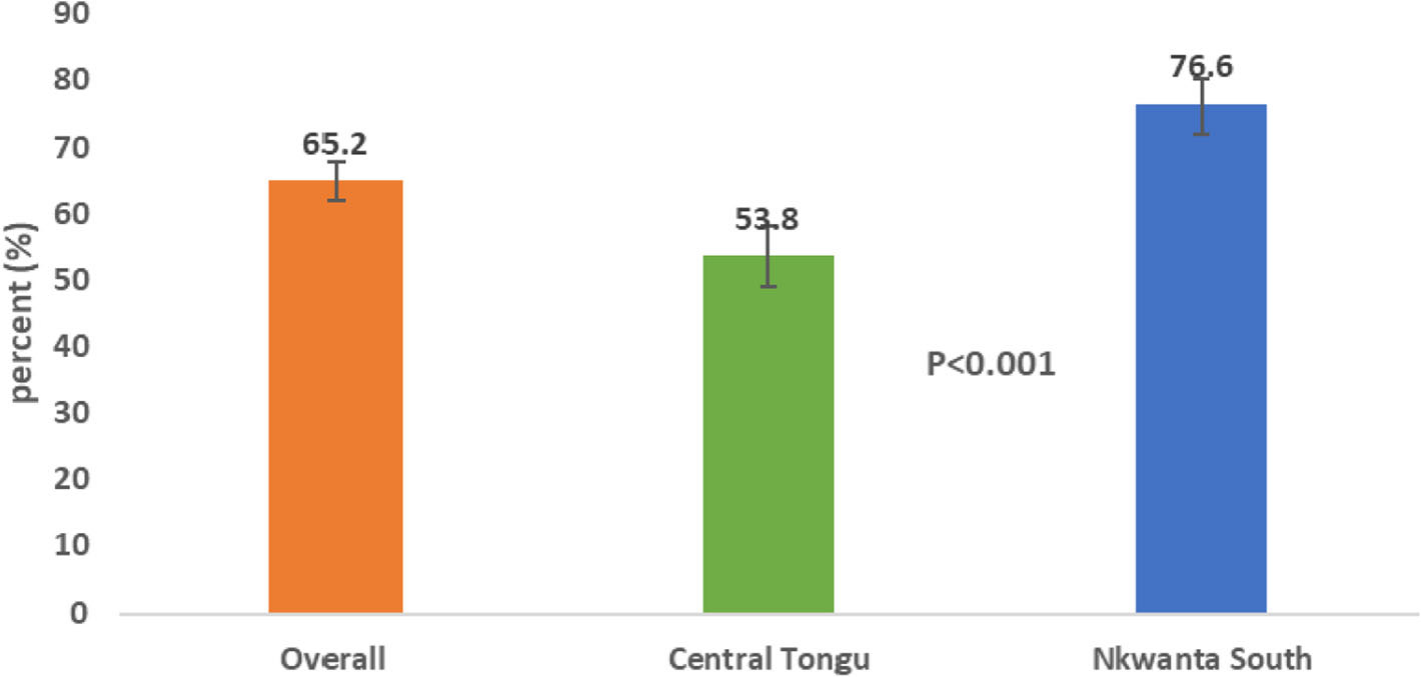
Level of utilisation of CHPS services overall and by district

Figure 3 presents the level of utilisation of CHPS as an aggregation of all the seven utilisation variables. Overall, the level of utilisation of CHPS was 65.2%. While the level of utilisation of CHPS was 76.7% in Nkwanta South, it was 53.8% in Central Tongu.

### Influence of socio-demographic characteristics on the utilisation of CHPS

Table 2 presents the association between socio-demographic characteristic and the odds of the utilisation of CHPS. Respondents in Nkwanta South Municipality were 3.17 times significantly [95%CI = 3.98–9.76, *p* < 0.001] more likely to utilise CHPS compared with those in the Central Tongu District. Females were 1.75 times significantly [95%CI = 1.27–2.39, *p* < 0.01] more likely to utilise CHPS than males. Respondents aged 30–39, 40–49 and 50 years and above were 18% [AOR = 0.82, 95%CI = 0.54–1.26], 36% [AOR = 0.64, 95%CI–0.40] and 51% [AOR = 0.49, 95% CI = 0.31–0.79] respectively less likely to utilise CHPS compared with those aged less than 30 years.

**Table 2:** Influence of socio-demographic characteristic and the utilisation of CHPS

Variables	Utilisation		Chi-square (***p***-value)	COR (95% CI) ***p***-value	AOR (95% CI) ***p***-value
	Low Utilisation	High Utilisation			
**District**			57.80(< 0.001)		
Central Tongu	233(66.4)	271(41.3)		Ref.	Ref.
Nkwanta South	118(33.6)	386(58.7)		2.81 (2.14; 3.68) < 0.001	3.17 (2.35; 4.25) < 0.001
**Sex**			41.67(< 0.001)		
Male	202(57.6)	239(36.4)		Ref.	Ref.
Female	149(42.4)	418(63.6)		2.37 (1.81; 3.08) < 0.001	1.75 (1.27; 2.39) 0.001
**Age group**			8.06(0.045)		
< 30	95(27.1)	201(30.6)		Ref.	Ref.
30–39	83(23.7)	187(28.5)		1.06 (0.74; 1.51) 0.729	0.82(0.54; 1.26) 0.370
40–49	73(20.8)	129(19.6)		0.83 (0.57; 0.57) 0.349	0.64 (0.40; 1.00) 0.053
50+	100(28.4)	140(21.3)		0.66 (0.46; 0.46) 0.022	0.49 (0.31; 0.79) 0.003
**Marital status**			21.48(< 0.001)		
Married/Co-habiting	265(75.5)	552(84.0)		Ref.	Ref.
Divorced/Separated	11(3.1)	27(4.1)		1.17 (0.57; 2.41) 0.653	1.29 (0.61; 2.78) 0.499
Widowed	15(4.3)	28(4.3)		0.89 (0.47; 1.71) 0.739	0.91 (0.45; 1.85) 0.794
Never married/ never Co-habited	60(17.1)	50(7.6)		0.40 (0.26; 0.59) < 0.001	0.50 (0.30; 0.83) 0.007
**Education**			42.15(< 0.001)		
None	98(27.9)	188(28.6)		Ref.	Ref.
Primary	57(16.2)	140(21.3)		1.28 (0.57; 2.41) 0.218	1.53 (0.99; 2.36) 0.052
JHS	100(28.5)	247(34.6)		1.28 (0.57; 2.41) 0.142	0.65 (1.12; 2.43) 0.011
SHS	65(18.5)	67(10.2)		0.53 (0.35; 0.82) 0.004	0.77 (0.46; 1.26) 0.296
Tertiary	31(8.9)	15(2.3)		0.25 (0.12; 0.48) < 0.001	0.39 (0.13; 1.22) 0.077
**Occupation**			36.79(< 0.001)		
Unemployed	31 (8.8)	44 (6.7)		Ref.	Ref.
Trader	39 (11.1)	100 (15.2)		1.81 (1.00; 3.25) 0.049	1.27 (0.65; 2.49) 0.475
Farming	199 (56.7)	427 (65)		1.51 (0.93; 2.46) 0.098	1.45 (0.81; 2.64) 0.211
Trace Person	36 (10.3)	30 (4.6)		0.58 (0.30; 1.14) 0.118	0.49 (0.22; 1.08) 0.078
Seamstress/Hairdresser	14 (4.0)	37 (5.6)		1.86 (0.86; 4.01) 0.113	1.01 (0.43; 2.36) 0.976
Civil Servant	32 (9.1)	19 (2.9)		0.41 (0.20; 0.86) 0.019	1.10 (0.37; 3.32) 0.854
**NHIS Status**			10.65(< 0.01)		
Non-subscriber	213(60.7)	328(49.9)		Ref.	Ref.
Subscriber	138(39.3)	329(50.1)		1.54 (1.18; 2.01) 0.001	1.51 (1.22; 2.03) 0.318

Respondents who were never married were 50% significantly less likely [AOR = 0.50, 95%CI = 0.30–0.83, *p* < 0.01] to utilise CHPS than those who were married. Those who attained SHS or tertiary educational levels were 23% [AOR = 0.77, 95%CI = 0.64–1.26] and 61% [AOR = 0.39, 95% CI = 0.13–1.22] respectively less likely to utilise CHPS as compared with those with no formal education and. Subscribers to the NHIS were, however, 1.51 times more likely to utilise CHPS compared with those who were non-subscribers [95% CI = 1.22–2.03].

## Satisfaction with CHPS

**Fig. 4:**
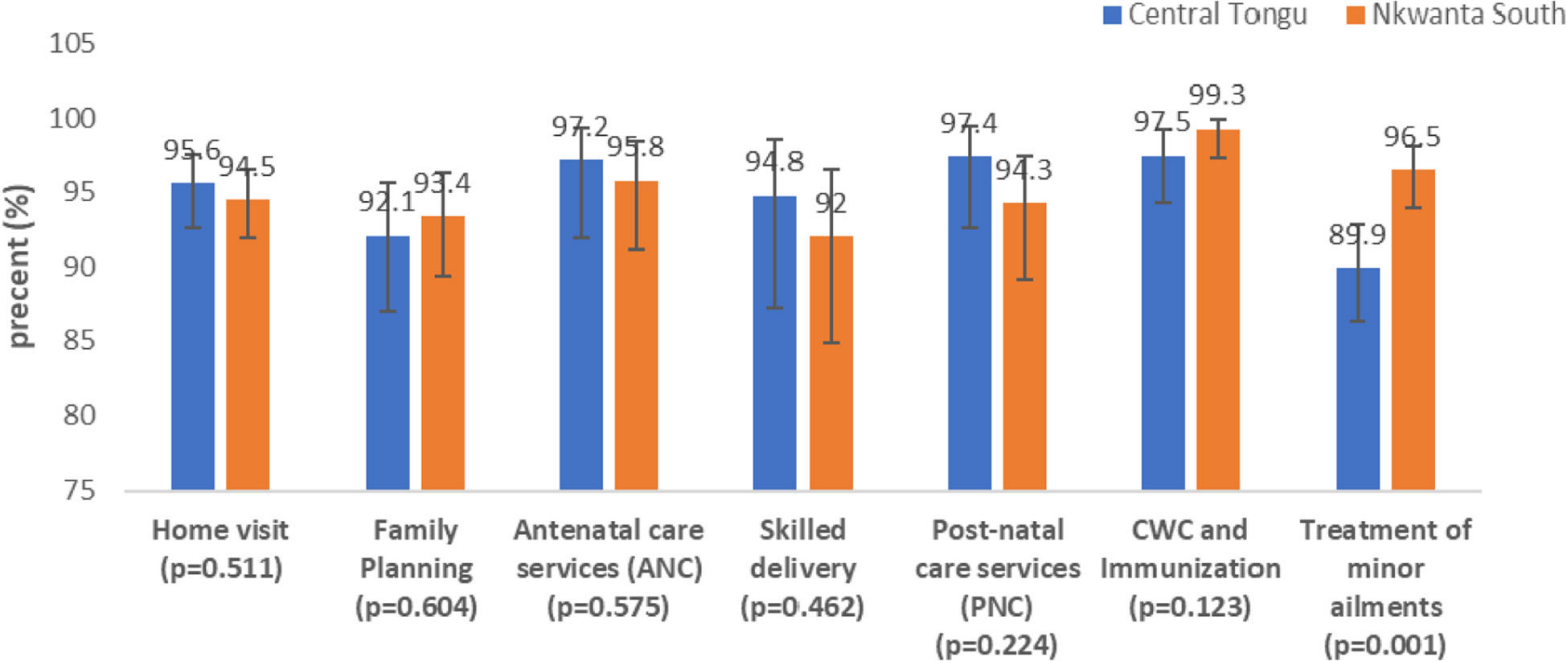
Satisfaction with various CHPS services by district

Figure 4 presents respondents’ satisfaction with the various CHPS services by district. Satisfaction with the treatment of minor ailments was significantly higher in Nkwanta South than Central Tongu (*p* < 0.01). No significant differences were, however, observed in the satisfaction of respondents with home visit, FP, ANC, skilled delivery, PNC as well as CWC and immunisation between the two SLDs.

**Fig. 5:**
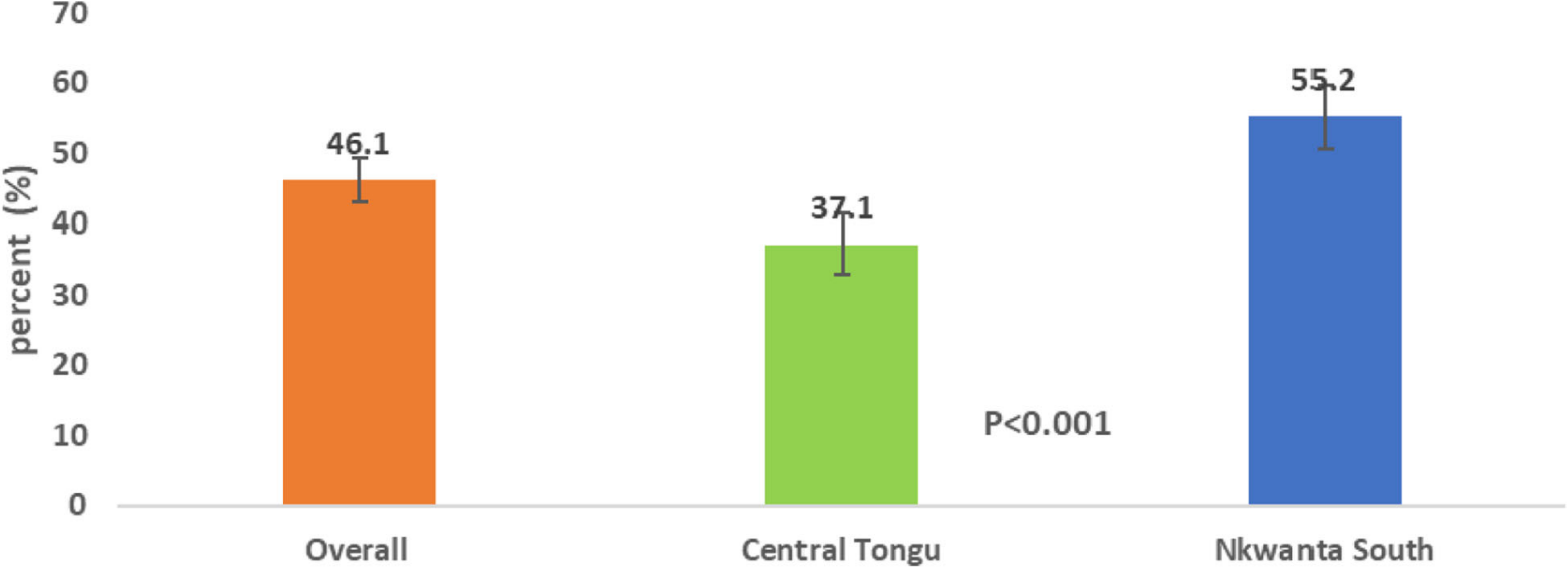
Level of satisfaction with CHPS services overall and by district

Figure 5 presents the level of satisfaction with CHPS as an aggregation of all the satisfaction variables. Overall, the level of satisfaction with CHPS was 46.1%. While the level of satisfaction with CHPS was 55.2% in Nkwanta South, it was 37.1% in Central Tongu.

### Influence of socio-demographic characteristic and utilisation of CHPS on satisfaction with CHPS

Table 3 presents the influence of socio-demographic characteristic and utilisation of CHPS on satisfaction with CHPS. Community members in Nkwanta South Municipality were 2.77 times [95%CI = 1.56–4.90, *p* < 0.001] more likely to be satisfied with CHPS services compared with those in the Central Tongu District. Respondents who were aged 50 years and above were 3.10 times more likely to be satisfied with CHPS services compared with those aged less than 30 years [AOR = 3.10, 95% CI = 1.25–7.68), *p* = 0.014]. Females and civil servants were 53% [AOR = 0.47, 95%CI = 0.25–0.90, *p* < 0.05] and 99.6% [AOR = 0.04, 95%CI = 0.0–0.67, *p* < 0.05] less likely to be satisfied with CHPS services compared with males and those who were unemployed respectively.

**Table 3:** Influence of Socio-demographic characteristics and utilisation on satisfaction with CHPS

Variables	Level of Satisfaction		Chi-square (χ^**2**^) (***p***-value)	COR (95%CI) ***p***-value	AOR (95%CI) ***p***-value
	Not satisfied [***N*** = 543] n (%)	Satisfied [***N*** = 465] n (%)			
**District**			33.05(< 0.001)		
Central Tongu	317(58.4)	187(40.2)		Ref.	Ref.
Nkwanta South	226(41.6)	278(59.8)		2.08(1.62, 2.68) < 0.001	2.77(1.56, 4.90) < 0.001
**Sex**			67.35(< 0.001)		
Male	302(55.6)	139(29.9)		Ref.	Ref.
Female	241(44.4)	326(70.1)		2.93(2.26, 3.81) < 0.001	0.47(0.25, 0.90) 0.023
**Age Group**			18.70(< 0.001)		
< 30	140(25.8)	156(33.6)		Ref.	Ref.
30–39	139(25.6)	131(28.1)		0.84(0.60, 1.17) 0.320	0.88 (0.39, 1.99) 0.767
40–49	107(19.7)	95(20.4)		0.79(0.55, 1.14) 0.214	1.99 (0.81, 4.89) 0.132
50+	157(28.9)	83(17.9)		0.47(0.33, 0.67) < 0.001	3.10 (1.25, 7.68) 0.014
**Marital Status**			33.91(< 0.001)		
Married/Co-habiting	406(74.8)	411(88.4)		Ref.	Ref.
Divorced/Separated	22(4.1)	16(3.4)		0.71(0.37, 1.38) 0.325	0.69 (0.20, 1.99) 0.566
Widowed	32(5.9)	11(2.4)		0.33(0.16, 0.68) 0.002	0.53 (0.16, 1.70) 0.293
Never married	83(15.2)	27(5.8)		0.32(0.20, 0.50) < 0.001	1.63 (0.58, 4.59) 0.352
**Educational level**			43.37(< 0.001)		
None	162(29.8)	124(26.7)		Ref.	Ref.
Primary	91(16.8)	106(22.8)		1.52(1.05, 2.19) 0.024	1.26 (0.56, 2.79) 0.570
JHS	161(29.6)	186(40.0)		1.50(1.10, 2.06) 0.010	1.24 (0.63, 2.48) 0.526
SHS/	89(16.4)	43(9.2)		0.63(0.40, 0.97) 0.037	0.74 (0.27, 2.03) 0.563
Tertiary	40(7.4)	(1.3)		0.19(0.08, 0.47) < 0.001	8.40(0.52, 133.95) 0.132
**Occupation**			46.09(< 0.001)		
Unemployed	44(8.1)	31(6.7)		Ref.	Ref.
Trader	61(11.2)	78(16.8)		1.81(1.02, 3.20) 0.040	1.08 (0.32, 3.65)0.898
Farmer	318(58.6)	308(66.2)		1.37(0.84, 2.23) 0.199	1.12(0.37, 3.36) 0.830
Tradesman	50(9.2)	16(3.4)		0.45(0.21, 0.93) 0.033	0.46 (0.09, 2.20) 0.333
Seamstress/hairdresser	25(4.6)	26(5.6)		1.47(0.72, 3.02) 0.286	0.25 (0.04, 1.47) 0.126
Civil servant	45(8.3)	6(1.3)		0.18(0.07, 0.49) 0.001	0.04 (0.0, 0.67) 0.026
**NHIS Status**			12.20(< 0.001)		
Non-subscriber	319 (58.7)	222(47.7)		Ref.	Ref.
Subscriber	224 (41.3)	243(52.3)		1.55(1.21, 2.00) < 0.001	1.45 (0.53, 1.68) 0.872
**Home visit**			129.94(< 0.001)		
No	241(44.4)	54(11.6)		Ref.	Ref.
Yes	302(55.6)	411 (88.4)		6.07 (4.36, 8.45) < 0.001	27.47 (11.51, 65.54) < 0.001
**Family Planning**			374.78(< 0.001)		
No	476 (87.7)	129 (27.7)		Ref.	Ref.
Yes	67 (12.3)	336 (72.3)		18.50 (13.34, 26.65) < 0.001	23.54(11.97, 46.29) < 0.001
**Antenatal care services**			332.70(< 0.001)		
No	533 (98.2)	225 (48.4)		Ref.	Ref.
Yes	10 (1.8)	240 (51.6)		56.85 (29.63, 109.07) < 0.001	52.30(14.56, 187.77) < 0.001
**Skilled Delivery services**			240.70(< 0.001)		
No	541 (99.6)	290 (62.4)		Ref.	Ref.
Yes	2 (0.4)	175 (37.6)		163.23(40.20, 662.65) < 0.001	3.92 (0.27, 56.45) 0.314
**Postnatal care services**			320.25(< 0.001)		
No	528 (97.3)	223 (48.0)		Ref.	Ref.
Yes	15 (2.7)	242 (52.0)		38.19(22.16, 65.84) < 0.001	9.47(2.87, 31.19) < 0.001
**CWC and Immunisation**			453.93(< 0.001)		
No	457 (84.2)	79 (17.0)		Ref.	Ref.
Yes	86 (15.8)	386 (83.0)		25.96 (18.58, 36.26) < 0.001	25.95(12.71, 53.00) < 0.001
**Treatment for minor ailments**			(< 0.001)		
No	214 (39.4)	63 (13.6)		Ref.	Ref.
Yes	329 (60.6)	402 (86.4)	84.07	4.15 (3.02, 5.69) < 0.001	15.76 (7.22, 34.43) < 0.001

Respondents who utilised home visit, family planning, antenatal care services were also 27.47 [95%CI = 11.51–65.54, *p* < 0.001], 23.54 [95%CI = 11.97–46.29, *p* < 0.001] and 52.30 [95%CI = 14.56–187.77, *p* < 0.001] times respectively more likely to be satisfied with CHPS services compared with those who did not utilise those services. Respondents who utilised PNC, CWC and immunisation, as well as services related to the treatment of minor ailment were 9.47 [95%CI = 2.87–31.19, *p* < 0.001], 25.95 [95%CI = 12.71–53.00, *p* < 0.001] and 15.76 [95%CI = 7.22–34.43, *p* < 0.001] times more likely to be satisfied with CHPS services compared with those who did not utilise them respectively.

## Discussion

This study examined community utilisation and satisfaction with CHPS services in two system learning districts of the CHPS+ Project in Ghana. We found that the level of utilisation of CHPS services in the two SLDs of our study was lower than the 73% reported by Wiru et al. [15] in the Kintampo North Municipality of Ghana. Comparatively, we realised in our study that the level of utilisation was higher in the Nkwanta South Municipality than in the Central Tongu District. Community members in Nkwanta South Municipality were more likely to utilise CHPS services compared with those in the Central Tongu District. This finding may be because Nkwanta South was the first district to pilot CHPS in the Volta Region after the Navrongo experiment in 1999 [23]. Therefore, community members might have been used to CHPS. Besides, the comparatively poorer road network in Nkwanta South coupled with the wider geographical space the municipality covers, makes it difficult for many communities to access health services at the Nkwanta South Municipal Hospital and the St. Joseph’s Hospital which are both located at Nkwanta, the Municipal Capital [17, 24]. Meanwhile, CHPS is provided in virtually all the communities of the municipality thus becomes the readily utilised health service for the people.

While Central Tongu is located in the southern coastal part of the Volta Region, Nkwanta South is situated at the Northern savannah belt [19]. Our finding of lower utilisation of CHPS services in Central Tongu may be related to previous findings that people in the southern coastal areas of Ghana usually experience a multiplicity of healthcare facilities and services [25]. Their perception of the quality of services including CHPS, therefore, easily becomes negative due to the awareness that they can always change their service providers/health facilities whenever they feel dissatisfied with services utilised [26]. The lower level of utilisation realised in the Central Tongu District has negative implications for Ghana’s attainment of UHC which all developing countries are required to achieve by the year 2030 [27].

We found that females were more likely to utilise CHPS services than males. This is similar to findings from Kintampo where females were 6 times more likely to use CHPS services than males [15]. The possible reasons could be that apart from family planning services, most CHPS services such as ANC, CWC, PNC, and skilled delivery, are female-focused. Additionally, research has shown that females by nature exhibit better health-seeking behaviour than males and this may be due to gender role differentiations including social class, education, and vulnerability to ill-health [28].

Our findings show that NHIS subscribers were more likely to utilise CHPS services than non-subscribers. This might be due to the financial protection which the scheme offers its subscribers [28, 29, 30]. This is because once subscribers pay affordable annual premiums, they can utilise health services covered by the scheme without paying anything at the point of service delivery [26, 29]. Also, indigents, pregnant women, Social Security and National Insurance Trust (SSNIT) pensioners and active contributors, as well as children younger than 18 years do not pay the annual premiums for subscribing to the scheme [30, 31]. All these groups of people in the community are, thus, likely to find it easier utilising CHPS services than those who have to pay out of pocket to utilise such services due to their non-subscription status.

The level of satisfaction recorded in our study was lower than the 65.8% satisfaction level of CHPS services recorded by Wood and Esena [16] in the Komenda Edina Eguafo Abrem Municipality.

While the level of satisfaction was high in Nkwanta South, it was low in Central Tongu. The variation in the level of satisfaction observed between the two districts illustrates the role of geographical antecedents discussed earlier and the deplorable nature of roads in the Nkwanta South Municipality which restrict community members’ access to CHPS and the fact that they are not able to have alternatives to CHPS services [32].

Our study also found that females were less likely to be satisfied with CHPS services than males. The possible reasons could be that as the main users of CHPS services such as family planning, ANC, CWC, PNC and skilled delivery they are more likely to encounter the challenges associated with CHPS implementation. This finding is congruent to the postulation by Azha et al. [33] that females were less likely to be satisfied with primary health care services such as CHPS.

### Strengths and limitations

Our study is the first effort at understanding the levels of community utilization and satisfaction with CHPS in Ghana. This is a strength because, even though CHPS was implemented to address the health needs of Ghanaian residents by bringing health care to the doorsteps of communities, there have not been studies examining the extent to which this goal has been achieved. Our findings are, therefore, essential in informing policy directions regarding community ownership of CHPS implementation. A limitation of our study is the fact that we depended on verbal reports of community members. The accounts given by the study participants regarding their utilization and satisfaction with CHPS services might have, therefore, suffered response and recall biases.

## Conclusion

While the level of CHPS utilisation is high, the level of satisfaction is low. The utilisation and satisfaction with CHPS are higher in Nkwanta South than in Central Tongu. Ghana may not be able to achieve the goals of universal health coverage by the year 2030 if current levels of utilisation and satisfaction with CHPS services persist. To accelerate progress towards the achievement of UHC with CHPS as the vehicle through which primary health care is delivered, we recommend that community members especially males should be given more public education on the relevance of CHPS by the GHS to increase their utilisation of CHPS among them. District/municipal/metropolitan and regional health directorates in Ghana should implement interventions geared towards improving the quality of CHPS services to increase community satisfaction. The GHS and the NHIA should sensitise communities as well as institute innovative strategies including extensive public education to increase subscription to the NHIS since it has implications for CHPS service utilisation and satisfaction.

## Supplementary information

**Additional file 1.**

## Data Availability

The datasets used and/or analysed during the current study available from the corresponding author on reasonable request.

## Abbreviations

ANC: Antenatal Care
AOR: Adjusted Odds Ratio
CHO: Community Health Officer
CHPS: Community-Based Health Planning and Services
COR: Crude Odds Ratio
CWC: Child Welfare Clinic
FP: Family Planning
GEHIP: Ghana Essential Health Interventions Programme
GHS: Ghana Health Service
LMICs: Low- and Middle-Income Countries
NHIA: National Health Insurance Authority
NHIS: National Health Insurance Scheme
PHC: Primary Health Care
PNC: Postnatal Care
SDG: Sustainable Development Goal
SLDs: System Learning Districts
SPH: School of Public Health
UN: United Nations
UHAS: University of Health and Allied Sciences
UDS: University for Development Studies
UHC: Universal Health Coverage
WHO: World Health Organisation
