# Supplementary material for: Community utilisation and satisfaction with the community-based health planning and services initiative in Ghana: a comparative study in two system learning districts of the CHPS+ project
Source: BMC Health Serv Res. 2020 Sep 9;20:845. doi: 10.1186/s12913-020-05678-5 (PMC7488145; doi:10.1186/s12913-020-05678-5)
Supplement: Supplementary file 1 — Additional file 1. [file 12913_2020_5678_MOESM1_ESM.doc]

**UNIVERSITY OF HEALTH AND ALLIED SCIENCES**

**SCHOOL OF PUBLIC HEALTH (CHPS+ PROJECT)**

**QUESTIONNAIRE ON CHPS IMPLEMEMENT IN GHANA**

Name of Interviewer**: _________________________________________**

Interview ID: ­­­­­­­­­­­­­­­_____

Date of interview (DD/MM/YY): / _/_____

Time of interview: Start ___________End____________

District: Community:

| **100: SOCIO-DEMOGRAPHIC CHARACTERISTICS**  Fill in or circle the appropriate responses for questions 101 to 109 | | |
| --- | --- | --- |
| **No** | **Variable** | **Attributes** |
| 101 | Sex | 1. Male……………………………………………..……1 2. Female ………………………………………….……2 |
| 102 | Age (In completed years) | ­­­­­­­­­­­­­­­­­­­­­­__________________ |
| 103 | Religion | 1. Christianity……………………...……..……...…..….1 2. Islam…………………………………..….........…..…2 3. African Traditional………………….…….............….3 4. Other (specify)………………………………… |
| 104 | Marital status? | 1. Married or living together……………..…………..…1 2. Divorced/Separated…………………..…….…….…..2 3. Widowed……………………………..……………....3 4. Never married and never lived together……………...4 |
| 105 | Ethnicity | 1. Ewe…………………………………………………...1 2. Akan……………………………………....………….2 3. Guan……………………………………..……….…..3 4. Ga-Dangme………………………………............…..4 5. Mole-Dagbani…………………………………….….5 6. Other (specify) ___________________________ |
| 106 | Highest educational level | 1. No formal education………………………………….1 2. Primary……………………………………………….2 3. Junior High School (JSS/JHS)………………………3 4. SHS/Vocational/A’level/O’level……………………4 5. Tertiary………………………………………………5 |
| 107 | Main occupation | __________________________ |
| 108 | Average monthly income | __________________________ |
| 109 | NHIS Status | 1. Active subscriber…………………………..……..1 2. Non-subscriber……………………………….…..2 |

| **200: CHPS SERVICES UTILIZATION AMONG COMMUNITY MEMBERS**  Thank you for your participation so far. Now, I would like us to discuss services which you received from CHOs in the past 6 months  For questions 201 to 208, kindly indicate whether you have used the following services rendered by CHOs within **the past 6 months**  **(Tick [√] all that apply)** | | |
| --- | --- | --- |
| **No** | **Service** |  |
| 201 | Home visit |  |
| 203 | Family Planning |  |
| 204 | Antenatal care services (ANC) |  |
| 205 | Skilled delivery |  |
| 206 | Post-natal care services (PNC) |  |
| 207 | Child Welfare Clinic and Immunization |  |
| 208 | Treatment for minor ailments/first-aid |  |

| **300: SATISFACTION WITH SERVICES**  Now, we would talk about your satisfaction with the services which you received from the CHOs in the past 6 months  For the following questions (301-308), kindly indicate your level of satisfaction with the services which you received from the CHOs (as indicated in 200) | | | | | |
| --- | --- | --- | --- | --- | --- |
| **No** | **Service** | **Level of satisfaction** | | | |
|  |  | **Very satisfied** | **Satisfied** | **Not satisfied** | **Not at all satisfied** |
| 301 | Home visit |  |  |  |  |
| 303 | Family Planning |  |  |  |  |
| 304 | Antenatal care services (ANC) |  |  |  |  |
| 305 | Skilled delivery |  |  |  |  |
| 306 | Post-natal care services (PNC) |  |  |  |  |
| 307 | Child Welfare Clinic and Immunization |  |  |  |  |
| 308 | Treatment for minor ailments/first-aid |  |  |  |  |

Thank you for participating in this study
